# Deficits in early neural tube identity found in CHARGE syndrome

**DOI:** 10.7554/eLife.01873

**Published:** 2013-12-24

**Authors:** Parthiv Haldipur, Kathleen J Millen

**Affiliations:** 1 **Parthiv Haldipur** is in the Center for Integrative Brain Research, Seattle Children’s Research Institute, Seattle, United Statesparthiv.haldipur@seattlechildrens.org; 2 **Kathleen J Millen** is in the Center for Integrative Brain Research, Seattle Children’s Research Institute, Seattle, United States and Division of Genetic Medicine, Department of Pediatrics, University of Washington, Seattle, United Stateskathleen.millen@seattle.childrens.org

**Keywords:** Cerebellar malformation, cerebellum, CHARGE syndrome, CHD7, FGF8, OTX2/GBX2, Human, Mouse

## Abstract

Long predicted from studies of model vertebrates, the first human example of abnormal patterning of the early neural tube leading to underdevelopment of the cerebellum has been demonstrated.

**Related research article** Yu T, Meiners LC, Danielsen K, Wong MTY, Bowler T, Reinberg D, Scambler PJ, van Ravenswaaij-Arts CMA, Basson MA. 2013. Deregulated FGF and homeotic gene expression underlies cerebellar vermis hypoplasia in CHARGE syndrome. *eLife* **2**:e01305. doi: 10.7554/eLife.01305

**Image** MRI scan of a patient with CHARGE syndrome

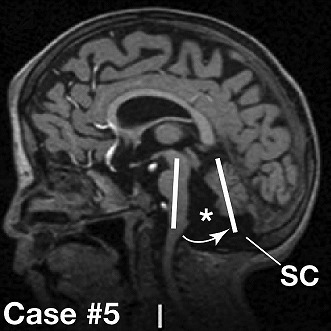

CHARGE syndrome is a genetic condition that involves multiple malformations in newly born children. The acronym stands for coloboma (a hole in the eye), heart defects, choanal atresia (a blockage of the nasal passage), retarded growth and development, genital abnormalities, and ear anomalies (Lalani et al., 2012). Now, in *eLife*, Albert Basson of King’s College London (KCL) and co-workers—including Tim Yu of KCL as first author—report that underdevelopment of a region of the cerebellum called the vermis is also associated with CHARGE syndrome (Yu et al., 2013).

Most cases of CHARGE syndrome can be related to a mutation in the gene that encodes CHD7, a protein that is involved in remodelling chromatin (Janssen et al., 2012). The latest work by Yu et al. shows that loss of CHD7 also disrupts the development of the early neural tube, which is the forerunner of the central nervous system. This results in underdevelopment (hypoplasia) of the cerebellar vermis. This finding is extremely exciting as it represents the very first example of this particular class of neural birth defect to be observed in humans.

One of the first steps in the development of the central nervous system is the establishment of gene expression domains that segment the neural tube into the regions that become the forebrain, the midbrain, the hindbrain and the spinal cord. Subsequently, signalling centres establish the boundaries between these regions and secrete growth factors which pattern the adjacent nervous tissue (Kiecker and Lumsden, 2012). The best understood signalling centre is the Isthmic Organizer, which forms at the boundary of the midbrain and the hindbrain. This centre secretes fibroblast growth factor 8 (Fgf8) and other growth factors, and is essential for defining the regions of the neural plate that will become the posterior midbrain and the cerebellum (Figure 1).

**Figure 1.**
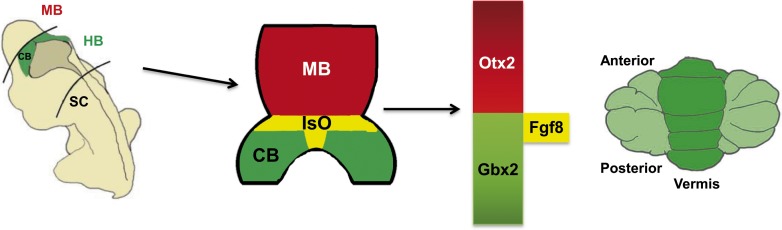
The role of the Isthmic Organizer. The establishment of gene expression domains along the anterior-posterior axis helps to segment the developing brain into the forebrain, midbrain (MB), hindbrain (HB) and spinal cord (SC). Signalling centres established at the boundaries between these segments secrete growth factors which pattern the adjacent nervous tissue. The Isthmic Organizer (IsO; shown in yellow) forms at the boundary of the posterior midbrain and anterior hindbrain: the IsO secretes Fgf8 and other growth factors, and is essential for defining the regions of the neural plate that will become the posterior midbrain (shown in blue) and the cerebellum (CB; green). The homeobox genes *Otx2* and *Gbx2* are involved in the formation of the IsO and in regulating the expression of Fgf8 by the IsO. Mutations in the CHARGE syndrome gene, *CHD7*, can alter *Otx2*, *Gbx2* and *Fgf8* expression, resulting in underdevelopment of a region of the cerebellum called the vermis (right).

Figure modified from Basson and Wingate, 2013

Extensive experiments in model vertebrates (such as mice, chickens and zebrafish) have shown that if the Isthmic Organizer fails to develop, there is dramatic and rapid cell death in adjacent regions of the neural tube. Mutant mice in which the Isthmic Organizer cannot express Fgf8 at all do not survive post-natally (Chi et al., 2003). However, reduced Fgf8 signalling or a failure to maintain Fgf8 expression does not result in animal death, but rather causes cerebellar vermis hypoplasia (Basson et al., 2008; Sato and Joyner, 2009). The genetic regulatory network that leads to the formation of the Isthmic Organizer is highly conserved across vertebrates, and beyond (Robertshaw and Kiecker, 2012), and it has long been postulated that disruption of the Isthmic Organizer must, therefore, be a cause of human cerebellar malformation. However, until now, this classical developmental phenotype had not been recognized in human patients.

With recent developments in neuroimaging, neuropathology and neurogenetics, many developmental disorders of the cerebellum, including cerebellar vermis hypoplasia, have emerged as causes of neurodevelopmental dysfunction (Doherty et al., 2013). Together, these disorders are relatively common, occurring roughly once in every 3000 live births. Cerebellar malformations can occur in isolation or as part of a broader malformation syndrome involving multiple systems. Although several cerebellar malformation genes have been identified, no gene directly involved in the formation or function of the Isthmic Organizer had previously been implicated in this class of birth defect, suggesting that Isthmic Organizer disorders might not be compatible with survival in humans.

Previously several clinical reports had noted cerebellar deficits in a few CHARGE syndrome patients. Now Yu, Basson and co-workers—who are based in London, Groningen and New York—report the results of MRI scans of a large cohort of 20 patients with CHARGE syndrome (caused by a mutation in *CHD7*; Yu et al., 2013). They confirm multiple cerebellar abnormalities in 55% of these patients, with 25% of the patients having cerebellar vermis hypoplasia.

Yu et al. also report the results of experiments of mice lacking one or both working copies of the *Chd7* gene. Mice lacking one working copy of the gene did not have any obvious cerebellar phenotype, but cerebellar vermis hypoplasia was evident when one copy of the *Fgf8* gene was also removed, suggesting genetic synergy between *Chd7* and *Fgf8*. Expression of *Fgf8* and its downstream gene *Etv5* were significantly down regulated at the junction of the midbrain and the hindbrain during the early development of *Chd7* mutant mice. Thus, reduced Fgf8 expression represents an underlying cause of CHARGE-related vermis hypoplasia because cerebellar development is exquisitely sensitive to the level of Fgf8 signalling.

Going one step further, Yu et al. next demonstrated that the expression of two genes that regulate Fgf8 expression at the Isthmic Organizer (*Otx2* and *Gbx2*) is altered in mice lacking both working copies of the *Chd7* gene. Chromatin immunoprecipitation assays subsequently revealed that Chd7 is associated with enhancer elements belonging to the *Otx2* and *Gbx2* genes, which suggests that *Chd7* has a direct role in regulating these essential Isthmic Organizer genes.

Together these results provide substantial evidence that cerebellar vermis hypoplasia in CHARGE syndrome is caused by dysfunction of the Isthmic Organizer in the early embryo. Further, the work of Basson, Yu and co-workers suggests that Isthmic Organizer disruption may be a more common cause of human cerebellar malformation than previously thought. Although complete loss-of-function mutations in genes that are central to the formation of the Isthmic Organizer (such as *Otx2*, *Gbx2* and *Fgf8*) are still likely to be incompatible with human life, this study implies that genes which regulate the expression of central Isthmic Organizer genes may cause cerebellar malformation when mutated.

CHD7 is expressed in a wide variety of tissues during development, and CHARGE syndrome phenotypes indicate that it has tissue-specific and developmental stage-specific roles. A difficult question remains as to how CHD7 achieves different functions in different tissues. For example although loss of *Chd7* disrupts Isthmic Organizer *Fgf8* expression, *Fgf8* expression is normal in the adjacent pharyngeal arches of the embryo. One possibility is that Cdh7 has different binding partners in different tissues.

Intriguingly, it has recently been shown that Cdh7 interacts with a small number of other transcription factors in neural stem cells, and that mutations in these other factors underlie a number of human malformation disorders (Engelen et al., 2011). One exciting prediction of this finding in combination with the work of Yu et al. on CHD7, is that systematic analysis of cerebellar morphology may uncover previously unrecognized cerebellar deficits related to Isthmic Organizer function in a wide range of other human birth defect syndromes.
